# Four Chemical Trends Will Shape the Next Decade's Directions in Perfluoroalkyl and Polyfluoroalkyl Substances Research

**DOI:** 10.3389/fchem.2018.00103

**Published:** 2018-04-05

**Authors:** Matthias Kotthoff, Mark Bücking

**Affiliations:** Department Environmental and Food Analysis, Fraunhofer Institute for Molecular Biology and Applied Ecology, Schmallenberg, Germany

**Keywords:** per- and polyfluoroalky substances, PFAS, analytical chemistry, consumer products, PMT substances, PBT substances

## Abstract

Per- and polyfluoroalkyl substances (PFAS) represent a versatile group of ubiquitously occurring chemicals of increasing regulatory concern. The past years lead to an ever expanding portfolio of detected anthropogenic PFAS in numerous products encountered in daily life. Yet no clear picture of the full range of individual substance that comprise PFAS is available and this challenges analytical and engineering sciences. Authorities struggle to cope with uncertainties in managing risk of harm posed by PFAS. This is a result of an incomplete understanding of the range of compounds that they comprise in differing products. There are analytical uncertainties identifying PFAS and estimating the concentrations of the total PFAS load individual molecules remain unknown. There are four major trends from the chemical perspective that will shape PFAS research for the next decade.

1. Mobility: A wide and dynamic distribution of short chain PFAS due to their high polarity, persistency and volatility.
2. Substitution of regulated substances: The ban or restrictions of individual molecules will lead to a replacement with substitutes of similar concern.
3. Increase in structural diversity of existing PFAS molecules: Introduction of e.g., hydrogens and chlorine atoms instead of fluorine, as well as branching and cross-linking lead to a high versatility of unknown target molecules.
4. Unknown “Dark Matter”: The amount, identity, formation pathways, and transformation dynamics of polymers and PFAS precursors are largely unknown.

These directions require optimized analytical setups, especially multi-methods, and semi-specific tools to determine PFAS-sum parameters in any relevant matrix.

## Introduction

Per- and Polyfluoroalkyl Substances (PFAS) have been a challenging subject of technical and scientific disciplines for the last decades. And they will be for the next. PFAS represent a vast group of different molecules with a great variety of applications and physicochemical properties. Key characteristic is their strong C-F bond, the resulting inertness, and stability. When scanning scientific search engines for the keyword “perfluorinated” several thousand hits for scientific contributions arise (PubMed: 2292; Web of Science: 8396). This huge investigative pressure does not only show the scientific interest in the topic, but also the economic, societal and political relevance. For 2017 only, Web of Science identified 575 hits, which indicates ongoing research actions. PFAS-research is influenced by many contributing factors and perspectives, such as the variable amounts of fluorinated substances produced. Furthermore, their commercial and environmental transport routes across the globe–as pure industrial chemicals or as fabrics - or in trace amounts on consumer products play a role. Moreover, the used substance portfolio is influenced constantly by improved production technologies, legal status of individual compounds, design of new molecules with optimized characteristics, new fields of application, and the availability of new research methods. A couple of recent reviews specify current analytical methods (Ruan and Jiang, 2017), substance spectra (Xiao, 2017), and resulting environmental challenges (Shi and Cai, 2014; Krafft and Riess, 2015). The relevance of PFAS research is underlined by multiple adverse effect routes that are described upon human PFAS exposure (Bach et al., 2016; Ballesteros et al., 2017).

From the literature published in the past years, from discussions and presentations, four major trends can be drawn for PFAS-related research needs. So far, the main perfluorinated structural elements used for the production of water repellent materials were based on chain lengths (the carbon atom backbone) above six. This led to an abundance of C8-based perfluoroalkylic acids (PFCAs), especially perfluorooctane sulfonic acid (PFOS) and perfluorooctanoic acid (PFOA). Manufacture and use of PFOS was prohibited in the EU from 2006 (2006/122/EC) and is listed in Annex B of the Stockholm convention. PFOA has been regulated in 2017 to phase out in 2020, and was nominated a candidate for Annex B of the Stockholm convention. The efficiency of such ban was shown, as it already led to a decreasing trend of PFOS in human blood serum samples in a retrospective study from 2011 (Schröter-Kermani et al., 2013).

## Mobility

The phase out and ban of specific substances did not change principals of PFAS chemistry, but let to an increased usage of “alternatives,” including short chain PFAS with chain lengths below six carbon atoms. However, besides these developments, short chain PFCAs did not receive the same scientific and public attention. Today, we know that short chain PFCAs are produced and emitted in significant amounts, e.g., for use in formulations replacing the C8 based chemistry (Wang et al., 2016). Thus more attention will be directed to substances such as trifluoroacetic acid (TFA), perfluoropropanoic acid (PFPrA), perfluorobutanoic acid (PFBA), and perfluorobutane sulfonic acid (PFBS). In addition to PFBA and PFBS which sources are known and their use is intentional, several studies have shown the worldwide occurrence of TFA and also PFPrA in aqueous environmental samples (Wujcik et al., 1997, 1998, 1999; Scott et al., 2006a,b; Taniyasu et al., 2008; Kwok et al., 2010; Yeung et al., 2017). While it is suspected that the abundance of TFA originates from HFC-1234yf (2,3,3,3-tetrafluoropropene) and HFC-134a (1,1,1,2-tetrafluoroethane) used as cooling agents in mobile air conditioners (Henne et al., 2012; Russell et al., 2012), the origin of PFPrA is yet unknown (Yeung et al., 2017). The key difference between long chain and short chain PFAS is their mobility: While long chain PFAS strongly absorb to solid mater, such as soil, which renders them practically immobile, short chain PFAS show a high mobility *via* water bodies and leachates, and can thus be detected in ground water samples (Gellrich et al., 2012). Moreover, very short chain PFAS are also volatile and can be distributed within atmospheric routes and enter water cycles and other environmental compartments in remote regions far from areas of direct exposure (Hu et al., 2013; Scheurer et al., 2017). These factors, promote recent efforts to shift the focus toward mobility as a key chemical parameter of concern, besides the established criteria persistent (P), bioaccumulative (B), and toxic (T) toward persistent mobile organic chemicals (PMOCs) (Reemtsma et al., 2016) and persistent, mobile (M), or toxic (PM or PMT) (Arp et al., 2017; Neumann et al., 2017). The future importance is to recognize the “M” criterion to be more than the reciprocal “B” criterion. The property of a substance not being “B” does not automatically induce mobility and, moreover, mobility may cause environmental and health concerns even exceeding those of the “B” criterion.

The importance of the mobility criterion is becoming increasingly clear considering environmental damages caused by a number of accidental or intentional incidents. Wang et al. (2017) mentioned incidents in Australia, Germany, Norway, Sweden, United Kingdom, and the United States. Severe issues are dealt with in the German states of North Rhine-Westfalia (Lanuv, 2017) and Baden-Wuerttemberg (Regierungspräsidium Karlsruhe, 2017). The wide-spread impacts to ground and drinking water with long chain PFAS result in the need for soil remediation, while the short chain PFAS cause the need for drinking water treatment, and shutdown of several drinking water supplies.

The toxicity of only a few PFAS compounds has been investigated and safe PFAS exposure levels are not commonly agreed upon. Short chain PFASs are considered to be of similar concern and as persistent as long chain PFAS, but may have shorter half lives in organisms (Olsen et al., 2007, 2009). However, their toxicity is still not well understood. Therefore, according to the precautionary principle the use of short chain PFAS e.g., as alternatives for long chain PFAS should be avoided.

## Substitution of regulated substances

While for PFOA and PFOS concentrations in human samples show declining trends, this is not the case for other substances (Land et al., 2017). A restriction in the use of short chain PFASs, as described above, not only leads to a shift in the observed chain lengths but also to a diversification of the used molecules. By engineering the molecular structure, substances that exhibit very similar or even more desired characteristics than PFOA and PFOS, but are not regulated and not yet integral part of authority initiated monitoring studies can be synthesized. Typical structural components are ether functions (Perfluoropolyethers, PFPEs), single positions that are chlorinated instead of fluorinated, or branched molecules (Wang et al., 2013; Rotander et al., 2015). The currently most popular substitutive for PFOA is probably 3H-perfluoro-3-[(3-methoxy-propoxy)propanoic acid] (ADONA), a PFPE. ADONA has already been detected in a variety of environmental samples (Heydebreck et al., 2015) and also recently in plasma of German blood donors (Fromme et al., 2017). Other prominent emerging substances are perfluoro-2-propoxypropanoic acid (PFPrOPrA), known as GenX, a technical mixture of chlorine substituted perfluoro sulphonic acid ethers, known as F53-B, and hydrogen substituted polyfluoroalkyls, like 2,3,3,3-tetrafluoro-2-(1,1,2,2,3,3,3-heptafluoropropoxy)propanoic acid (HFPO-DA) (Heydebreck et al., 2015). To cope with the dynamic industrial novelty, the scientific community will quickly have to adopt analytical methods and monitoring programs which need to include these compounds into the target lists. Besides the newly designed substitutes, PFAAs (perfluoroalkyl acids) and PFAS of varying chain lengths and mixtures of those are of course also candidates to replace banned and regulated chemicals. Despite the ban of PFOS and the regulation of PFOA, similar unbranched substances, and also uneven chained PFASs remain unaffected and are found in the environment, consumer products and human samples (Joyce Dinglasan-Panlilio et al., 2014; Zafeiraki et al., 2014; Kotthoff et al., 2015; Yeung et al., 2017). These are especially perfluorobutane sulphonic acid (PFBS), perfluoroheaxane sulphonic acid (PFHxS), perfluorobutanoic acid (PFBA), perfluorohexanoic acid (PFHxA), perfluoroheptanoic acid (PFHpA), and perfluorononanoic acid (PFNA). The efficiency of such singular regulatory measures is thus questionable. However, it can be an initiation to promote and support the efforts regulating PFAS subclasses as a whole. It's notable that PFHxS and PFOA, “their salts and related substances” have recently been listed as candidates for the Stockholm convention (Stockholm_Convention, 2017). The definition of the mentioned related substances will be interesting, as to whether this may also include molecules such as ADONA, GenX or F53-B, or at least pave the way to regulate them in the near future.

## Increase in structural diversity of existing PFAS molecules

A recent study identified as many as 2060 PFASs that are or were marketed for intentional applications, and that there are at least 3000 PFASs on the global market [KEMI (Swedish Chemicals Agency), 2015]. Many of these commercial products, and technical grade chemicals may contain unintended by-products in significant amounts (Wang et al., 2017).

Even well-known individual substances may appear as mixtures of several specific isomers. In the case of PFOS and PFOA, for example, major part of the whole relevant substance are the linear molecules, but also up to 10 branched isomers may account for the total amount of PFOS and PFOA. In contaminated ground water, these branched isomers account for up to 35% of the total long chain homologs (Pellizzaro et al., 2017). From studies with biosolids-amended soils, it was recently reported that the plant uptake and translocation for branched isomers is even higher than for the linear isomers. This renders the branched isomers a research subject with individual hazards on their own (Zhang et al., 2018). Many chemical and biochemical reactions, such as microbially mediated bio-transformations in soil and groundwater (Benskin et al., 2013) will lead to the formation of more unknown intermediate PFAS, such as the fluorotelomer sulphonates (Liu and Mejia Avendano, 2013), and persistent terminal transformation products. Using modern analytical techniques such as high resolution mass spectrometry in non-targeted and suspect screening approaches are leading to the discovery of more and more individual molecules (Rotander et al., 2015; Baduel et al., 2017; Newton et al., 2017; Xiao et al., 2017; Xu et al., 2017). Is this, because better and more sensitive instruments and analytical methods are available? Only in parts: a study of PFAS levels in German and Chinese human serum and blood samples indicates an increase in unidentified organofluorine compounds after the year 2000 (Yeung and Mabury, 2016). The authors analyzed the same set of retrospective samples with a specific and an unspecific Extractable Organic Fluorine (EOF) method (see below). This indicates an increase in human exposure toward new and unidentified PFASs that is irrespective of analytical techniques.

## Unknown “dark mater”

As the structural diversity of produced and marketed PFASs increases and many degradable polymers add to the complexity of the substance pattern, the amount of structures that are not captured with established analytical methods increases. Besides specific research oriented analytical methods for individual molecules or specific subgroups, few routinized off-the-shelf methods exist. Current analytical multi methods rely on (ultra) high performance liquid chromatography coupled to tandem mass spectrometry [(U)HPLC-MS/MS] and capture hardly more than 30 PFAS in one run. These methods usually detect C4 to C10 PFAA and C4, C6, and C8 PFSA, as they are also included in the present DIN guidance documents for water and sludge (DIN, 2011a,b). Recently published methods cover also precursor substances and substitutes that are extractable with conjoint methods. A draft ISO method for water actually aims to include 31 substances and will yet be extended with some target molecules (ISO, 2017). Its final validation would at least lead to these roughly 30 substances beingroutinely captured, and in future methods also for more complicated matrices than water, e.g., soil, blood or serum, and plant materials.

This analytical limitation does not allow to get a fast overview of the degree of exposure or contamination in terms of quantity and quality. In environmental incidents, even the identification of sources by means of the substance pattern may take valuable time. The complex mixture of substances used in aqueous film forming foam (AFFF) used as fire extinguishing foams, but also in other industrial branches is composed of numerous individual derivatives of PFCAs and PFSAs and other PFASs.

All the unknown substances and those not captured with the applied analytical methods will not be accounted for and balanced and can, consequently, not be considered for health and environmental assessments. Moreover, since there are numerous and diverse sources of PFAS, the nature of molecules released into the environment are not known and not part of commercial or academic methods, and consequently incidents of PFAS release can go undetected or will at least be underestimated. To cope with this gap, various non-specific methods were established allowing to estimate the total load of PFAS.

One method is to determine the sum of extractable or absorbable organic fluorine compounds (EOF or AOF; Yeung et al., 2008). This analytical method, based on Combustion Ion Chromatography, is just being standardized. However, the individual PFAS cannot be differentially determined and so, the EOF or AOF can only be used as an orientation value and support the specific analysis using targeted methods such as UHPLC-MS/MS. Another sum parameter is the determination of total fluorine using Particle-Induced Gamma Ray Emission (PIGE) Spectroscopy (Hashiguchi et al., 2013). This method is suited to generate a fast overview of the total fluorine load, but similar to EOF, AOF, and PIGE does not allow for the speciation of chain lengths and structural details. The disadvantage of both PIGE and EOF/AOF is that the detection limits are in the μg/L range, whereas regulatory standards are now increasingly at ng/L levels.

The determination of Total Oxidizable Precursors (TOP) is an alternative, which involves the oxidation of all precursors present in samples during sample preparation to form PFCAs and PFSAs, which can then be quantified by conventional analysis (Houtz and Sedlak, 2012). Analysis of the sample before and after this oxidative pretreatment allows for a quantitative estimate of the precursor content of the sample. This method can provide limits of detection in the 1–2 ng/L range, as seen with conventional analysis for PFAAs using LC-MS/MS. Moreover, the resulting PFCAs (and less frequently observed the PFSAs) partly reflect the structural composition of the precursors. However, with fluorotelomers this does not occur in a manner which necessarily preserves the chain length of the PFAAs evolved after the oxidative digest. Thus, the resulting PFAAs may be shortened by one or two carbon atoms. This chain shortening effect is however analogous to the natural partial defluorination and shortening exhibited due to microbial attack of PFAAs (Liu and Mejia Avendano, 2013). The measurement of slightly shorter chain length PFAAs from the perflouroalkyl groups in the fluorotelomer precursors, still allows a stoichiometric estimate of the precursor concentrations. Then, as shorter chain PFCAs are generally less bioaccumulative and thus can be excreted more quickly than longer chain, their toxicity is generally diminished. An assumption that the slightly shorter chain PFAAs are representative of the toxicity of the slightly longer chain fluorotelomer precursors will therefore not be overestimated. One difference between the PFAAs formed from the TOP assay and those occurring via natural biotransformation is that the TOP assay tends to generate PFCAs from sulphonamide precursors, whereas micro-organisms generate PFSAs. However, with the sulphonamide precursors the TOP assay digest retains the perflouroalkyl chain length of the precursors, as also seen with microbial attack. This may only be relevant for risk assessment purposes when considering the C6 PFAAs and PFHxA is classed as short chain with diminished bioaccumulation potential compared to PFHxS, which is classed as long chain. However, to be able to determine whether the PFHxA generated after TOP assay of a sulphonamide precursor would occur as PFHxS in the environment, it should be possible to measure the branched chain PFHxA after TOP assay. This will provide a good indication of whether the precursor was a sulphonamide [synthesized via electrochemical fluorination (ECF)] or a fluorotelomer (made via fluorotelomerisation), as only ECF generates branched chain isomers. Branched chain isomers were typically present at 30–35% of straight chain (Benskin et al., 2010; Pellizzaro et al., 2017) so the concentration of the branched PFHxA could be multiplied by 3.3 to give an estimate the concentration of the PFHxA that should be assessed as if it is long chain PFHxS. These calculations should also consider that differential transport of branched vs. linear compounds is known to occur in many systems (Benskin et al., 2010, 2013), so the branched vs. linear composition is not typically preserved at exactly 30% in environmental samples. In order to use this method not only as a tool of approximation, but to assess potential toxicity and thus form part of a regulatory framework to manage risks from PFASs, these pragmatic assumptions considering interpretation of TOP assay data could be adopted. Given the major advantages of using TOP assay it is seen as the most promising method for future improvements in environmental management of PFASs and could be considered as the analytical method of choice for future research projects.

## Conclusion

The growing knowledge and understanding of precursors indicates a potential presence in consumer products such as food packaging materials and outdoor clothing. Prior assessments of PFAAs in products may have failed to detect many unknown PFASs (Kotthoff et al., 2015). In order to be aware of realistic exposures and resulting risks, future work, also with respect to specific toxicological impacts, has to be directed to understanding both of PFAAs and their precursors and to answer some key questions: What are the more abundant PFASs, moreover, which are the abundant precursors and how quickly do they transform to the persistent PFAAs? How do mobile PFASs distribute from commodities into drinking water and produce? Which evolving analytical methods to measure total PFAS (including precursors) are most promising? What needs to be optimized to have cost effective and fast screening methods that provide a comprehensive assessment of PFASs, with output data that can be pragmatically integrated into environmental management strategies to allow risk based management of potential for harm?

These new, more comprehensive, analytical techniques make a huge stride in taking PFASs assessment forward. However, in agreement with Wang et al. (2017) for the foreseeable future, and unless safe and known alternatives are available, PFASs research will not be an ending story, as PFASs are here to stay for future millennia (Allen, 2018).

## Author contributions

MK had the idea for the manuscript and drafted it. MK and MB performed the literature research, wrote and revised the manuscript.

### Conflict of interest statement

The authors declare that the research was conducted in the absence of any commercial or financial relationships that could be construed as a potential conflict of interest.
